# Machine Learning Approaches for Early Identification of Subclinical Ketosis and Low-Grade Ruminal Acidosis During the Transition Period in Dairy Cattle

**DOI:** 10.3390/life15091491

**Published:** 2025-09-22

**Authors:** Samanta Arlauskaitė, Akvilė Girdauskaitė, Dovilė Malašauskienė, Mindaugas Televičius, Karina Džermeikaitė, Justina Krištolaitytė, Gabija Lembovičiūtė, Greta Šertvytytė, Ramūnas Antanaitis

**Affiliations:** Large Animal Clinic, Veterinary Academy, Lithuanian University of Health Sciences, Tilžės Str. 18, LT-47181 Kaunas, Lithuania; akvile.girdauskaite@lsmu.lt (A.G.); dovile.malasauskiene@lsmu.lt (D.M.); mindaugas.televicius@lsmu.lt (M.T.); karina.dzermeikaite@lsmu.lt (K.D.); justina.kristolaityte@lsmu.lt (J.K.); gabija.lemboviciute@lsmu.lt (G.L.); greta.sertvytyte@lsmu.lt (G.Š.); ramunas.antanaitis@lsmu.lt (R.A.)

**Keywords:** dairy cattle, machine learning, early detection, milk composition, innovative technologies

## Abstract

This study evaluated six supervised machine learning (ML) models for early detection of subclinical ketosis and low-grade ruminal acidosis in dairy cows during the transition period. Ninety-four Holstein cows were monitored for 21 days postpartum using in-line milk analyzers and intraruminal sensors that continuously recorded milk composition, behavioral, and physiological parameters. Based on clinical examination, blood β-hydroxybutyrate concentration, and fat-to-protein ratio, cows were classified as healthy (*n* = 44), subclinical ketosis (*n* = 24), or subclinical acidosis (*n* = 26). Among the tested models, Random Forest and XGBoost achieved perfect accuracy within this dataset, while Logistic Regression reached 89.5%, Decision Tree 84.2%, and both Naive Bayes and Support Vector Machine 78.9%. These results suggest that ensemble approaches, particularly Random Forest and XGBoost, show strong potential for integration with precision livestock technologies, but their apparent performance should be interpreted cautiously and confirmed in larger, multi-farm studies.

## 1. Introduction

The transition period in dairy cows, which encompasses the three weeks before and after calving, is a critical phase for maintaining animal health and optimizing productivity. During this time, cows undergo substantial physiological, metabolic, and endocrine adjustments to initiate and support lactation [1]. Proper management is essential, as this period is marked by a heightened risk of disease and represents the most pronounced negative energy and protein balances, both of which can negatively impact fertility and overall health [2].

In this context, precision monitoring has gained increasing importance with the advancement of technologies that enable automated herd management and continuous health surveillance. The integration of commercially available systems has improved the efficiency of milking, feeding, and behavioral monitoring, facilitating the early detection of health disturbances before clinical symptoms emerge [3]. As part of this advancement, automated herd management systems have been developed to assist farmers in monitoring individual animals within large herds. These technologies can track critical health parameters—such as estrus activity, body temperature, and rumen pH—offering valuable insights into each cow’s metabolic status and enabling the timely identification of potential health issues [4]. This proactive approach allows for timely implementation of preventive and therapeutic interventions, ultimately supporting better animal welfare and production outcomes [3].

Machine learning, a branch of artificial intelligence (AI), employs statistical methods to analyze large datasets for predicting cow performance or detecting disease events [5]. It is particularly well-suited for handling complex relationships and interactions that arise from the growing number of variables in modern dairy production systems [6]. Machine learning techniques are being increasingly utilized to improve the detection of subclinical ketosis in dairy cows, offering solutions to the limitations of conventional diagnostic approaches. In one study, a scoring system was created to evaluate the performance of various machine learning models in predicting cows at risk, using data from routine milk performance tests. Among the models tested, logistic regression demonstrated the highest effectiveness, achieving a sensitivity of 0.74 and specificity of 0.76 at specific β-hydroxybutyrate (BHB) thresholds. These findings highlight its potential as a practical tool for monitoring subclinical ketosis in dairy herds [7]. Expanding the application of machine learning, a recent study by Touil et al. [8] employed partial least squares, random forest (RF), and gradient boosting algorithms to predict reticuloruminal pH and subacute ruminal acidosis (SARA) based on mid-infrared spectral data from individual cow milk samples. Although the prediction of reticuloruminal pH showed limited accuracy (R^2^ rarely exceeding 0.12), the models demonstrated promising results for SARA classification, achieving up to 69% accuracy under nested leave-one-farm-out cross-validation [8]. These findings further support the integration of machine learning in precision livestock farming for early detection of metabolically driven disorders in dairy herds.

Our hypothesis was that automated monitoring systems, when combined with machine learning algorithms, can effectively identify early-stage metabolic disorders—specifically subclinical ketosis and low-grade ruminal acidosis—in dairy cows during the transition period by analyzing physiological, behavioral, and milk composition data.

The objective of this study was to evaluate the feasibility and accuracy of multiple supervised machine learning (ML) models in detecting early metabolic imbalances in dairy cows during the first 21 days postpartum. This was accomplished by integrating data from in-line milk analyzers and intraruminal sensors to classify cows into health-related groups based on fat-to-protein ratio (FPR), BHB concentration, and clinical examination findings. Notably, some of the models—particularly RF and extreme gradient boosting (XGBoost)—achieved perfect classification accuracy (100%).

## 2. Materials and Methods

### 2.1. Animals and Management

The study was conducted at the Practical Training and Research Center and Large Animal Clinic of the Lithuanian University of Health Sciences, located in central Lithuania, in Eastern Europe. The research period began on 2 October 2023, and continued through 30 June 2025.

The study involved 94 Holstein cows, including 48 first-lactation (primiparous) and 46 multiparous animals. Cows were maintained in a loose-housing system and received a total mixed ration (TMR) throughout the year. The ration was formulated to satisfy or exceed the nutrient requirements of a 550 kg Holstein cow producing 35 kg of milk per day. On a dry matter basis, the diet consisted of about 31% corn silage, 10% grass silage, 4% grass hay, 49% grain concentrate, and 6% mineral supplement. The chemical composition of the TMR was 50.7% dry matter, 28.3% neutral detergent fiber, 19.8% acid detergent fiber, 38.7% non-fiber carbohydrates, and 15.8% crude protein, with an energy density of 1.60 Mcal/kg of net energy for lactation. Feeding was provided twice daily at 08:00 and 16:00. Milking was performed with automated DeLaval robotic units (DeLaval Inc., Tumba, Sweden). The average live weight of the cows was 550 ± 45 kg. In 2024, the mean annual energy-corrected milk yield (4.1% fat, 3.4% protein) was 10,304 kg per cow.

### 2.2. Registration of the Parameters

In this study, milk composition (milk yield, fat, protein, lactose contents and FPR was continuously monitored using the Brolis HerdLine in-line milk analyzer (Brolis Sensor Technology, Vilnius, Lithuania), while rumination time, water intake, reticulorumen temperature, and activity levels were tracked using the SmaXtec monitoring system (SmaXtec Animal Care GmbH, Graz, Austria).

The daily milk composition data for each cow was calculated based on measurements from the Brolis in-line milk analyzer, which operates in the 2100–2400 nm spectral range and utilizes a GaSb-based, widely tunable external cavity laser spectrometer. This compact device, mounted directly on milking stalls or robotic milking units, monitored milk flow in transmission mode throughout the entire milking process. It required no reagents or routine maintenance. Milk composition data were captured every five seconds, and final values for fat and protein were calculated as flow-weighted averages across each milking session.

Reticulorumen parameters were recorded using SmaXtec boluses, which provide continuous, real-time monitoring to support animal health and welfare. Each bolus was administered orally by an experienced veterinarian using a dedicated applicator, following the manufacturer’s instructions. The boluses were gravity-settling and remained in the reticulum throughout the study.

Prior to administration, each bolus was activated, linked to the cow’s unique ear tag for individual identification, and registered in the central monitoring system. During administration, cows were restrained in self-locking head gates, and the bolus was placed at the base of the tongue. Animals were monitored for two hours post-administration to observe any adverse reactions.

Data collection was conducted via antennas connected to the SmaXtec system, which was equipped with a microprocessor, an analog-to-digital converter, and external memory storage. Data compilation and management were performed using SmaXtec Messenger software (version 4). Throughout the study, continuous measurements of reticulorumen temperature, rumination time, physical activity, and water intake were recorded. The dataset consisted of 13,680 daily observations collected from 94 Holstein cows via SmaXtec monitoring system. A detailed list of the measured variables, their corresponding units, and measurement intervals is provided in Table 1.

### 2.3. Grouping

This study targeted the transition period—specifically the first 21 days postpartum—during which cows undergo major metabolic adaptations. To assess the health status of each animal, a daily clinical examination was conducted at 9:00 a.m. by the same veterinarian throughout the study period. Concurrently, data on milk fat-to-protein ratio from the automatic milking system and blood BHB concentrations were recorded using MediSense and FreeStyle Optium H systems (Abbott, Maidenhead, UK) from capillary blood samples collected from the ear. All blood samples were obtained during routine clinical examinations. Based on their first 21 days of lactation mean fat-to-protein ratio (FPR), clinical examination information and BHB levels, cows were divided into three groups [9]:

Clinically healthy cows (group 0): (FPR 1.2–1.4; BHB < 1.2 mmol/L; *n* = 44), with no detectable signs of disease during examination;

Subclinical ketosis (group 1): (FPR > 1.4; BHB > 1.2 mmol/L *n* = 24);

Low grade ruminal acidosis (group 2): (FPR < 1.2; BHB < 1.2 mmol/L; *n* = 26), cows exhibited moderate to severe diarrhea with undigested feed particles in their feces—confirmed by sieving. Assessment for left-sided abomasal displacement was performed using percussion and auscultation of the left paralumbar fossa.

Only cows with complete datasets from both the in-line milk analyzers and intraruminal sensors were included in the study. Cows were eligible for exclusion if they exhibited clinical signs of disease (e.g., mastitis, lameness, displaced abomasum, metritis, or digestive disorders) or had incomplete sensor or analyzer data. However, no animals were excluded, as all cows met the inclusion criteria and remained clinically healthy throughout the observation period.

### 2.4. Model Development and Techniques

The records were exported to Microsoft Excel spreadsheets (Microsoft, 2021) and subsequently imported into the KNIME 5.4.4 analytics platform (KNIME GmbH, Konstanz, Germany) for further analysis. Prior to it, all datasets were inspected for completeness and quality. Records with missing or biologically implausible values were excluded. To facilitate model development and validation, the dataset was randomly partitioned into two subsets: 80% of the data were allocated to the training set and the remaining 20% to the testing set. This random division was performed using a pseudo-random number generator, ensuring that records were selected without systematic bias. A fixed random seed was applied during partitioning to guarantee reproducibility of the sampling procedure across analytical runs. For Random Forest, the maximum depth was limited to 10 and the number of trees set to 100. A fixed random seed was applied to ensure reproducibility. No systematic grid or random hyperparameter search was performed, which may have favored ensemble models such as RF and XGBoost.

After partitioning, 76 cows remained in the training dataset: 36 in Group 0, 18 in Group 1, and 22 in Group 2. The dataset was then used for model training. Six supervised classification algorithms were implemented for predictive modeling. The machine learning algorithms selected for this study included Logistic Regression, Decision Tree, Support Vector Machine (SVM), RF, Gradient Boosting, and Naïve Bayes. These models were chosen based on their distinct methodological strengths: Logistic Regression is a widely used statistical approach for classification tasks, particularly valued for its interpretability [10]. Decision Trees are commonly employed in data mining for constructing classification rules based on multiple predictors and for developing predictive models targeting specific outcomes [11]. SVM was included due to its well-established effectiveness in managing high-dimensional and imbalanced datasets, especially in binary classification scenarios [12]. RF, a robust ensemble method, is frequently used in modern machine learning applications due to its high accuracy and resistance to overfitting [10]. XGBoost are capable of capturing complex relationships, including latent interactions and higher-order effects that are difficult to model explicitly [13]. Naïve Bayes was incorporated for its foundation in Bayesian probability theory, offering a probabilistic approach to classification tasks [14]. The characteristics of each machine learning model are summarized in Table 2.

### 2.5. Performance Metrics

A confusion matrix was constructed for each classification task to evaluate model performance (Table 3). In these matrices, the rows represent the predicted classifications and the columns the actual (true) class labels. The elements were defined as follows: true positives (TP, diseased cows correctly classified), false positives (FP, healthy cows incorrectly classified as diseased), true negatives (TN, healthy cows correctly classified), and false negatives (FN, diseased cows incorrectly classified as healthy). Error (%) was calculated as the complement of accuracy, representing the overall misclassification rate. Cohen’s Kappa was used as a descriptive, chance-corrected measure of agreement between predicted and actual classifications, with 1.0 indicating perfect agreement and values above 0.75 generally considered substantial agreement.

From the confusion matrices, we obtained accuracy, sensitivity, specificity, precision, F1 score, and Matthews correlation coefficient (MCC). To provide estimates of uncertainty, 95% confidence intervals (95% CI) for sensitivity, specificity, precision, and accuracy were calculated using the Clopper–Pearson exact method for binomial proportions. All metrics were generated using the KNIME 5.4.4 analytics platform (KNIME GmbH, Konstanz, Germany).

### 2.6. Statistical Analysis

Descriptive statistics were computed using the KNIME 5.4.4 Analytics Platform (KNIME GmbH, Konstanz, Germany). Data distribution was evaluated from descriptive statistics prior to hypothesis testing. Variables with approximately normal distributions (milk yield, protein, lactose) were compared between groups using one-way ANOVA, while skewed variables (BHB, rumination time, fat) were analyzed with Kruskal–Wallis test.

### 2.7. Study Contribution and Design

**Figure 1:**
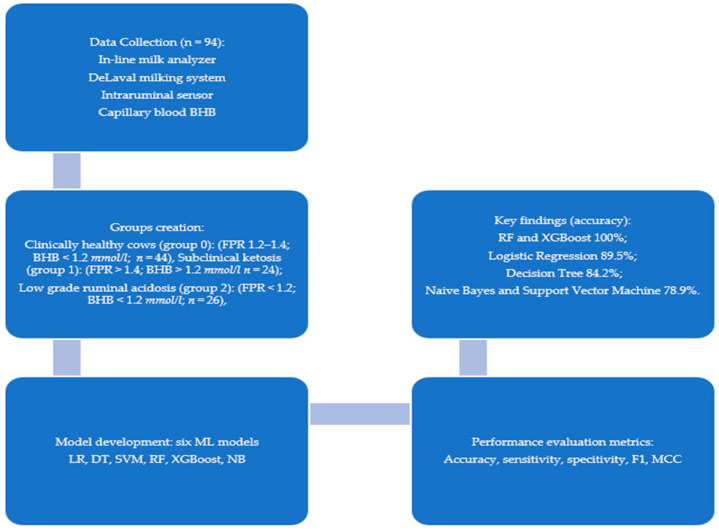
Study design and contributions.

This study contributes to precision dairy health management by evaluating the feasibility of six supervised machine learning models applied to automatically recorded milk, physiological, and behavioral data for the early detection of subclinical ketosis and low-grade ruminal acidosis in dairy cows. Unlike most previous studies, which typically relied on a single diagnostic indicator, we combined multiple data streams (blood BHB, fat-to-protein ratio, and clinical findings) to establish reference groups, thereby improving diagnostic robustness. The overall study design and key outcomes are summarized in Figure 1.

Key outcomes showed that ensemble methods (RF, XGBoost) reached perfect classification within this dataset, while traditional models such as Logistic Regression also performed well. These findings highlight both the potential of ML for integration with sensor-based herd monitoring and the need for cautious interpretation given the limited sample size.

## 3. Results

### 3.1. Performance Evaluation of Machine Learning Models

**Figure 2:**
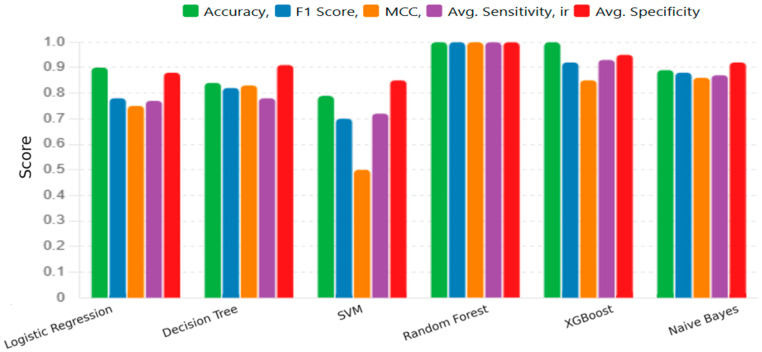
Model Performance Across Metrics: comparative performance analysis of six machine learning models for disease prediction. The models evaluated were Logistic Regression, Decision Tree, SVM, RF, XGBoost, and Naive Bayes. Performance was assessed using five metrics: Accuracy, F1 Score, MCC, Average Sensitivity, and Average Specificity. The *Y*-axis represents the score for each metric, on a scale from 0.0 to 1.0. The RF model demonstrated the highest performance, achieving near-perfect scores across all metrics.

**Figure 3:**
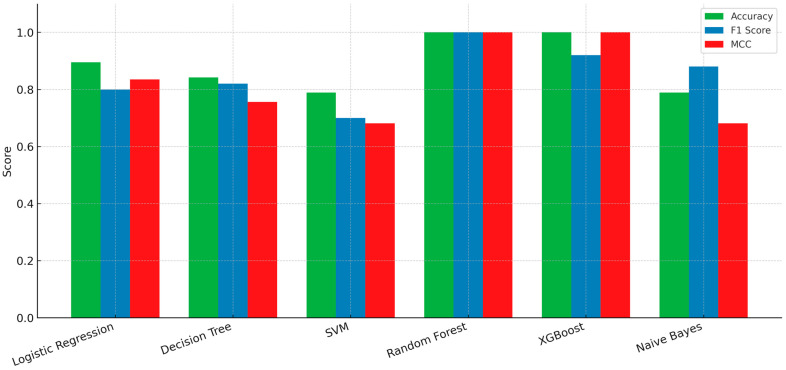
Comparative bar plot showing the accuracy, F1 score, and Matthews correlation coefficient (MCC) of six supervised machine learning models (Logistic Regression, Decision Tree, Support Vector Machine, Random Forest, XGBoost, and Naïve Bayes) for early detection of subclinical ketosis and low-grade ruminal acidosis in dairy cows.

This section provides a detailed assessment of six machine learning algorithms used to detect early signs of subclinical ketosis and low-grade ruminal acidosis in dairy cows. The diagnostic performance of each model: (Logistic Regression, Decision Tree, SVM, RF, XGBoost, and Naive Bayes)—measured by accuracy, sensitivity, specificity, and related evaluation metrics—is presented and comparatively analyzed in Figure 2. The scores for each metric range from 0.0 to 1.0, where 1.0 indicates a perfect score. The results indicate that the RF model exhibited superior performance, achieving near-perfect scores (approximately 1.0) across all five evaluation metrics. The Decision Tree and XGBoost models also demonstrated robust performance, with the majority of their metrics scoring above 0.9 and 0.84, respectively. The Naive Bayes model showed strong results as well, with high Accuracy, F1 Score (approx. 0.9), and a particularly high Average Specificity (approx. 0.95). With perfect scores on all metrics (Accuracy, F1 Score, Sensitivity, Specificity, and MCC), the Random Forest model demonstrated good classification ability and the best overall performance. In contrast, Logistic Regression yielded moderate performance; while its Average Specificity was high (approx. 0.88), its F1 Score, MCC, and Average Sensitivity were notably lower. The SVM model was the least effective among the models tested, characterized by an MCC score of approximately 0.5, indicating a lower quality of classification despite having a moderate average specificity. To complement these results, a comparative bar plot (Figure 3) was added to visualize accuracy, F1 score, and MCC across all six models. This representation highlights the superior performance of ensemble methods, particularly Random Forest and XGBoost, compared with more traditional approaches. Table 3 summarizes the number of classification mistakes each machine learning model made within three health-related groups of dairy cows: group 0 (clinically healthy), group 1 (subclinical ketosis), and group 2 (low-grade ruminal acidosis). Most of the misclassifications occurred in group 0, particularly for the Naive Bayes, SVM, and Decision Tree models, which incorrectly classified 3, 3, and 2 animals, respectively. In contrast, no model misclassified any animals from group 1, indicating consistent performance in identifying this condition across all algorithms. For group 2, one misclassification was made by the Decision Tree, Naive Bayes, Logistic Regression, and SVM models, suggesting moderate difficulty in accurately identifying this group. The RF and XGBoost models achieved perfect classification with zero errors across all groups, resulting in 100% accuracy and a Cohen’s kappa of 1.0. However, this may be influenced by the limited sample size and should be interpreted with caution. Logistic Regression performed well overall, with only two errors—one in group 0 and one in group 2—achieving nearly 90% accuracy and the highest kappa (0.835) among the non-ensemble methods.

### 3.2. Descriptive Statistics of Physiological, Behavioral, and Production Parameters

Table 4 presents a descriptive statistical summary of the key physiological, behavioral, and production parameters collected from the dairy cattle in the study. The analysis includes the minimum, maximum, 95% quantile, mean, and standard deviation for each variable.

The herd is characterized by a relatively young population, with the lactation number ranging from 1 to 4 and a mean of 1.947 (SD = 1.114). The body condition score (BCS) of the animals was generally consistent and within a healthy range, with a mean of 3.461 and a low standard deviation of 0.296. Milk yield showed considerable variation, as expected, with a mean of 11.347 kg (SD = 2.876) and a 95% quantile of 16.49 kg. The average milk composition included 4.82% fat, 3.749% protein, and 4.567% lactose.

Behavioral and physiological parameters measured by sensors also exhibited wide-ranging distributions. Water intake and activity levels were highly variable, with standard deviations of 25.728 L and 4.638 units, respectively. Rumination time averaged 458.841 min per day. Notably, the reticulorumen temperature, a measure of core body temperature, was tightly regulated, with a narrow range (38.52 °C to 39.64 °C) and a very small standard deviation (0.247). This contrasts with the broader range observed in the ambient temperature sensor.

The metabolic health indicators are of particular importance. The FPR had a mean of 1.299, with a 95% quantile of 1.598. The blood BHB levels, a direct indicator of subclinical ketosis, averaged 0.828 mmol/L. The maximum observed BHB value of 1.6 mmol/L and a 95% quantile of 1.52 mmol/L suggest the presence of cows experiencing subclinical ketosis within the study population, as these values exceed the commonly accepted threshold of 1.2 mmol/L. This distribution validates the dataset’s utility for investigating subclinical metabolic disease (Table 4).

## 4. Discussion

The purpose of this study was to assess the potential of different supervised ML models for the early detection of low-grade ruminal acidosis and subclinical ketosis during the transition period. Dairy cows’ health status was evaluated using a comprehensive method that combined capillary blood BHB concentrations with automatically recorded physiological, behavioral, and milk composition data. The initial hypothesis was validated by the results, which showed that ML models, specifically Random Forest and XGBoost, had high classification accuracy, sometimes reaching 100%.

The ability of logistic regression to efficiently analyze binary outcomes and evaluate predictive factors linked to disease makes it a popular technique in veterinary diagnostics, especially for detecting metabolic disorders like acidosis and ketosis. With only two misclassifications (one in the low-grade ruminal acidosis group and one in the healthy group), the logistic regression model in our study showed high specificity (88%), and overall accuracy of 89.5%. Even though there are not many studies on this subject, what is known about it shows how useful logistic regression models can be in this situation. According to studies, anomalies in activity levels, milk production, and rumination time can all be early markers of ketosis, and adding them to regression models increases the predictive accuracy of the results [15]. Furthermore, logistic regression is especially useful for quantifying multiple risk factors and combining them into a logical diagnostic framework because ketosis frequently co-occurs with other metabolic disorders, highlighting the condition’s multifactorial nature [16]. This is especially true when combining multiple data streams, such as rumen pH, blood chloride levels, or milk BHB [17]. The significance of logistic regression as an open, comprehensible, and flexible tool in contemporary precision dairy health monitoring systems is highlighted by this multifaceted approach.

In veterinary practice, where decision interpretability and transparency are essential for practical implementation, decision tree models are especially attractive. With three misclassifications (two in the healthy group and one in the acidosis group), the decision tree classifier in our study had an overall accuracy of 84.2% and an 90% specificity. By analyzing a number of parameters, decision trees have been effectively used to determine the risk factors linked to subclinical ketosis. For example, other researchers showed how decision tree algorithms can be used to forecast lactational milk yield, showing how this approach can help make the best decisions by exposing hidden trends in cow productivity and health. These models can be used in a similar way to diagnose ketosis by assessing factors that are known to affect the risk of ketosis, such as body condition scores, rumination time, and milk production [18]. Additionally, the relationship between rumination time and subclinical acidosis and ketosis was examined, indicating that these parameters may be important in decision tree models. Proactive management strategies are made possible by the decision-making framework’s ability to use real-time data, such as rumination time, which has been demonstrated to vary significantly before clinical diagnoses [19]. To create a diagnostic framework, decision tree approaches can also be used to analyze combinations of ruminal pH levels, feeding practices, and biochemical markers. Determining that blood enzyme activity can act as a biomarker for acidosis diagnosis and showing that decision trees can use biomarker datasets to increase diagnostic precision [20]. Rule-based decision tree systems have been successfully used to identify risk factors for dystocia in dairy cows. The hierarchical structure of these models allows for the integration of key predictive variables, which could similarly be applied to the identification of metabolic disorders such as acidosis [21]. By clearly visualizing the ways in which each factor influences a cow’s risk of metabolic problems, decision trees can improve comprehension and intervention tactics.

The SVM algorithm was among the least successful models in this study, despite being frequently used in classification tasks because of its capacity to handle complex and high-dimensional data. Compared to the majority of the other algorithms that were tested, it had a lower classification performance as measured by other metrics and an overall accuracy of 78.9%. In the past, SVM has demonstrated promise in the early detection of metabolic diseases in dairy cows. Research has shown that SVM can successfully categorize health status using on-farm parameters like feed intake and behavioral indicators as well as mid-infrared spectroscopy data of milk [15,22]. These results demonstrate the model’s applicability for identifying both low-grade ruminal acidosis and subclinical ketosis, particularly when there are intricate and non-linear interactions between physiological, nutritional, and biochemical factors. Additionally, a data-driven method for identifying cows at risk for metabolic imbalances has been provided by the integration of various datasets (from metabolic profiles to environmental conditions) using SVM models [23]. Although SVM did not perform better than other models in our study, its ability to handle challenging classification problems raises the possibility that it could be a useful part of combined diagnostic systems.

Of all the models that were evaluated, the Random Forest algorithm performed the best. Its high classification, precision and dependability were demonstrated by its 100% accuracy, sensitivity, specificity, F1 score, and Matthews correlation percentage. The ability of Random Forest algorithms to predict metabolic condition has been observed in recent studies. Researchers discovered that identifying cows at risk for ketosis was highly accurate when they used a diverse dataset that included a range of physiological and management factors [24]. This study demonstrated how useful RF is for analyzing vast amounts of data and identifying trends that point to metabolic stress. A Random Forest model also demonstrated accuracy of 98.25% in the classification of bovine events, including the diagnosis of metabolic disorders such as subclinical ketosis. Numerous health parameters were assessed in the study, and the high recall and precision rates confirmed its potential for prompt and precise health assessments on dairy farms [25]. By integrating automated activity monitoring data with traditional medical records, Random Forest has been acknowledged for its exceptional predictive performance in agricultural applications, especially in the diagnosis of health disorders in dairy herds, improving the accuracy of ketosis detection [26]. Advancements have also been made in the use of Random Forest for diagnosing acidosis, particularly low-grade ruminal acidosis. By examining physiological and environmental data, research demonstrated how machine learning methods, such as Random Forest, could accurately forecast the risk of metabolic diseases like acidosis [27]. According to their findings, a broad range of indicators can be assimilated by machine learning models, which can then be used to link them to metabolic health and direct nutritional management. Because Random Forest models are naturally suited to multivariate problems, they are unique among algorithms. They can handle high-dimensional, sizable datasets, which are frequently found in contexts involving animal health monitoring. Additionally, the models’ interpretability, which can determine importance scores for specific features, enables farm managers and veterinarians to comprehend the crucial elements influencing health outcomes [28]. This interpretability is essential for facilitating informed decision-making in veterinary practice.

In our study, the XGBoost algorithm also showed perfect classification accuracy. It was one of the most sophisticated ensemble techniques, using the gradient boosting principle to process complex data efficiently. All cows, whether healthy or afflicted with low-grade ruminal acidosis or subclinical ketosis, were accurately classified, yielding 100% sensitivity, specificity, and accuracy. Nevertheless, whether using this approach or another, such flawless results should be interpreted cautiously because they might be a reflection of the study’s small sample size. Furthermore, using indicators like milk yield, body condition score, and metabolic parameters, other studies have also shown excellent results when applying this model for the detection of health disorders in dairy cattle, including ketosis [25]. A modified version of XGBoost called XGBOD, which combines supervised and unsupervised learning, demonstrated improved sensitivity in detecting atypical cases like subclinical ketosis early on, when clinical signs are not yet readily apparent [29]. While there are currently few direct studies on the use of XGBoost in acidosis diagnosis, initial findings indicate that this technique can be successfully applied to large datasets for analysis, such as feed composition, ruminal pH, and health indicators [30]. This suggests that XGBoost may be able to detect low-grade ruminal acidosis, particularly when several risk factors are taken into account at once. More focused research is required to completely validate its use in veterinary medicine, especially for conditions like acidosis, even though the current studies offer preliminary evidence.

The accuracy of the Naive Bayes model, which is based on a straightforward probabilistic classification method, was 78.9% in our investigation. Although the acidosis group demonstrated perfect accuracy, the healthy animal group experienced the most misclassifications (3 mistakes), while the subclinical ketosis group experienced one misclassification (1 mistake). Combining this model with other algorithms in hybrid systems that capitalize on the advantages of several approaches may increase its efficacy even more [31]. Additionally, the precise evaluation of true positive and false positive classifications helps assess the reliability of this approach in veterinary practice and contributes to the early diagnosis of metabolic disorders [25].

The relationship between milk fat-to-protein ratio (FPR) and blood BHB concentration is central for defining subclinical ketosis in early lactation. Thresholds of FPR > 1.4 are frequently used as practical markers of negative energy balance, while blood BHB > 1.2 mmol/L is widely accepted as the biochemical cut-off for subclinical ketosis [9,15]. Previous studies have shown that these indicators only moderately agree: some cows may exceed the FPR threshold without elevated BHB, reflecting milk composition changes influenced by diet and stage of lactation, while others may show increased BHB despite normal FPR values [19]. This highlights the multifactorial nature of energy metabolism during the transition period and suggests that combining FPR and BHB provides a more robust diagnostic framework. In our study, both measures were applied together for group classification, which strengthens biological plausibility, but further research is needed to quantify their agreement and predictive value across different herds.

Based on automatically recorded indicators and blood BHB concentration, our study showed that supervised machine learning models can serve as useful tools for the early detection of subclinical ketosis and low-grade ruminal acidosis. Both sophisticated ensemble algorithms, such as Random Forest and XGBoost, and more traditional methods, including logistic regression and Naive Bayes, demonstrated promising classification performance. However, the very high accuracies observed, in some cases reaching 100%, should be interpreted with caution, as they likely reflect the small sample size and single-farm design rather than the inherent reliability of the models. Herd-specific management practices, environmental conditions, and genetic background may influence both disease prevalence and model performance, and thus the results may not fully generalize to other herds, breeds, or production systems. Moreover, the study did not include benchmarking against established diagnostic thresholds, such as serum BHB > 1.2 mmol/L or milk fat-to-protein ratio < 1.2, leaving the added value of machine learning compared with conventional diagnostics uncertain. Future research should therefore focus on larger and more diverse animal populations, incorporate head-to-head comparisons with standard diagnostic criteria, and extend validation across multiple farms with different management systems. Beyond predictive performance, the economic implications of adopting sensor-based ML systems are also highly relevant for practical farm management. While conventional diagnostic methods rely on repeated blood sampling or laboratory milk testing, which are labor-intensive, invasive, and costly, sensor-based monitoring integrated with machine learning offers continuous, non-invasive surveillance that may reduce labor demands, animal handling stress, and production losses. Although initial investment in sensors and data infrastructure can be substantial, earlier detection and improved herd health outcomes may render such systems more cost-effective in the long term. Future studies should therefore not only validate diagnostic accuracy but also quantify the economic benefits of ML-driven monitoring to support adoption in commercial dairy herds.

## 5. Conclusions

The use of six supervised machine learning models for the early detection of low-grade ruminal acidosis and subclinical ketosis during the transition period was assessed in this study. The models were found to be potentially useful tools for identifying metabolic disorders based on automatically recorded physiological, behavioral, and milk composition indicators as well as capillary blood BHB concentrations. The classification accuracy of Random Forest and XGBoost was 100% among all tested algorithms, whereas the other models’ performance varied between 78.8% and 89.5%. By efficiently processing real-time herd-level data gathered from precision monitoring systems, the models were able to provide prompt insights into the health status of the animals. Although the present findings indicate that supervised machine learning models hold promise for detecting subclinical ketosis and low-grade ruminal acidosis in early-lactation cows, these results should be interpreted as preliminary. The perfect classification accuracy achieved by RF and XGBoost is likely a reflection of model overfitting on a limited dataset. Also, the relatively small sample size, single-farm design, and absence of external validation limit the strength of the conclusions. Rather than confirming immediate field applicability, this study provides exploratory evidence that machine learning approaches merit further investigation. Larger, multi-site datasets and independent validation are needed before these models can be considered robust tools for routine herd health management.

## Figures and Tables

**Table 1:** Data from automated monitoring and milking systems.

Sensor	Trait	Measurement Interval	Unit
SmaXtec bolus (SmaXtec Animal Care GmbH, Graz, Austria)	Activity	10 min	Arbitrary units (a.u.) specific to SmaXtec’s internal algorithm.
	Water intake		L/day
	Temperature		°C
	Rumination time		min/day
	Reticulorumen temperature		°C
Brolis Herdline in-line milk analyzer (Brolis Sensor Technology, Vilnius, Lithuania)	Milk yield	Measurements were taken every 5 s throughout each milking.	kg/day
	Protein		%
	Fat		%
	Fat to Protein ratio		
	Lactose		%
DeLaval milking system (DeLaval Inc., Tumba, Sweden)	Body Condition Score	Throughout each milking	Score 1–5

**Table 2:** Key parameters of machine learning models.

Model	Type	Key Parameters	Description
Logistic Regression	Linear model	Maximal numbers of epochs: 100,000 Epsilon: 10 × 10^5^ Learning rate strategy: fixed Step size: 0.1 Prior: uniform variance: 0.1	Uses a linear decision boundary; effective for binary classification problems.
Decision Tree	Tree-based model	Maximum number of stored patterns for HiLite-ing: 10,000	Builds a flowchart-like structure to split data based on feature thresholds.
SVM	Non-linear Kernel-based	Overlapping penalty: 1.0 Polynomial Power: 3.0 Bias: 1.0 Gamma: 1.0	Constructs hyperplanes in high-dimensional space; can use linear or RBF kernels.
RF	Ensemble of trees	Tree Options: Split Criterion: Information Gain Ratio Limit number of levels (tree depth): 10 Minimum node size: 1 Forest Options: Number of models: 100 Use static random seed: 175337971207	Aggregates predictions from multiple decision trees to improve generalization.
Gradient Boosted	Ensemble of boosted trees	Missing value handling: XGBoost Use static random seed: 1753382549121	Sequentially builds trees to correct previous errors; highly accurate.
Naïve Bayes	Probabilistic model	Default probability: 0.0001 Minimum standard deviation: 0.0001 Threshold standard deviation: 0.0 Maximum number of unique nominal values per attribute: 20	Based on Bayes’ theorem; assumes independence between predictors.

**Table 3:** Confusion matrices of the applied models.

Model	Group 0 (Mistakes)	Group 1 (Mistakes)	Group 2 (Mistakes)	Total Mistakes	Accuracy (%)	Error (%)	Cohen’s Kappa
Decision Tree	2	0	1	3	84.211	15.789	0.756
Naive Bayes	3	0	1	4	78.947	21.053	0.681
Logistic Regression	1	0	1	2	89.474	10.526	0.835
RF	0	0	0	0	100.0	0.0	1.0
SVM	3	0	1	4	78.947	21.053	0.681
XGBoost	0	0	0	0	100.0	0.0	1.0

**Table 4:** Descriptive statistics of the dataset variables.

Name	Minimum	Maximum	95% Quantile	Mean	Standard Deviation
Lactation number	1	4	4	1.947	1.114
BCS	2.6	4.2	3.9	3.461	0.296
Milk Yield	6.1	20.87	16.49	11.347	2.876
Fat	3.65	7.12	5.966	4.82	0.621
Protein	2.8	4.55	4.27	3.749	0.304
Temperature	32.7	37.54	36.888	35.665	0.768
Water intake	33.67	160.04	156.958	118.089	25.728
Activity	1.89	38.96	17.304	7.948	4.638
Rumination time	334.91	582.63	524.076	458.841	48.272
Temperature reticulorumen	38.52	39.64	39.42	38.928	0.247
F:P	0.87	1.99	1.598	1.299	0.174
BHB	0	1.6	1.4	0.828	1.013
Lactose	4.09	4.8	4.772	4.567	0.144

## Data Availability

Data are contained within the article.

## Abbreviations

The following abbreviations are used in this manuscript:

AI	artificial intelligence;
BHB	β-hydroxybutyrate;
FP	False positives;
FPR	fat-to-protein ratio;
MCC	Matthews correlation coefficient;
ML	machine learning;
RF	random forest;
SARA	subacute ruminal acidosis
SVM	support vector machine;
TMR	total mix ration;
TN	True Negatives;
TP	True positives;
XGBoost	extreme gradient boosted;
